# The Histone Demethylase IBM1 Positively Regulates *Arabidopsis* Immunity by Control of Defense Gene Expression

**DOI:** 10.3389/fpls.2019.01587

**Published:** 2019-12-18

**Authors:** Ching Chan, Laurent Zimmerli

**Affiliations:** Department of Life Science and Institute of Plant Biology, National Taiwan University, Taipei, Taiwan

**Keywords:** *Arabidopsis*, innate immunity, epigenetic regulator, IBM1, bacteria, defense, chromatin

## Abstract

Epigenetic modifications involve complex and sophisticated control over chromatin states and DNA methylation patterns, which are important for stress tolerance in plants. While the identification of epigenetic modulating enzymes keeps growing, such as *MET1*, for CG methylation; *CMT3*, *DRM2*, *DRM3* for CHH methylation; and *IBM1*, *SUVH4* for CHG methylation; the molecular roles of these regulators in specific physiological functions remain obscure. In a mutant screen, we identified *IBM1* as a new player in plant immunity. The *ibm1* mutants were hyper-susceptible to hemi-biotrophic bacteria *Pseudomonas syringae*. Accordingly, bacteria-induced up-regulation of *PR1*, *PR2*, and *FRK1* defense markers was abolished in *ibm1* mutants. Consistently, at the chromatin level, these defense marker genes showed enrichment of the inactivation mark, H3K9me2; while the activation mark H3K4me3 was reduced in *ibm1* mutants. Immunoprecipitation of associated chromatin further demonstrated that IBM1 binds directly to the gene body of *PR1*, *PR2*, and *FRK1*. Taken together, these data suggest that IBM1 plays a critical role in modulating *Arabidopsis* immunity through direct regulation of defense gene expression. Notably, IBM1 maintains a permissive chromatin environment to ensure proper induction of defense genes under some biotic stress.

## Introduction

Epigenetic control of the plant immunity response provides plasticity for the dynamic regulation of emerging pathogens, and at the same time maintains genome stability to avoid the generation of genomic lesion (Fu and Dong, 2013; Espinas et al., 2016). Response to pathogen-associated molecular pattern (PAMP)- or pattern-triggered immunity (PTI) and effector-triggered immunity (ETI) involves extensive transcriptional reprogramming (Tsuda and Katagiri, 2010; Huang and Zimmerli, 2014). In general, the plant defense response against biotrophic pathogens is mediated by salicylic acid (SA)-dependent signaling, while signals for resistance to necrotrophs occur through the jasmonic acid/ethylene (JA/ET) pathway (Katagiri et al., 2002; Alvarez et al., 2010). Crosstalk between the two pathways are well balanced to ensure priority of defense against either biotrophic or necrotrophic attack. Depending on the concentration of SA and JA, the two pathways can work synergistically or antagonize each other (Koornneef and Pieterse, 2008; Bari and Jones, 2009; Vlot et al., 2009). Eventually, both signaling cascades converge at the expression of antimicrobial pathogenesis-related (PR) genes in local environment as well as in distal tissue for long-term protection. Moreover, local defense responses can trigger a second layer of protection at distal tissue to protect the rest of the plant from subsequent infection, this phenomenon is known as systemic acquired resistance (SAR) (Fu and Dong, 2013).

Recent studies on epigenetic modifications and chromosome architecture brought novel insights beyond the concept of latent-state immune signaling component. “Immune memory” can last for weeks, months, the whole season, or even be passed on to progenies (Luna et al., 2012; Fu and Dong, 2013; Singh et al., 2014). In general, loss of DNA methylation makes plants more resistant to bacterial infection. For example, mutants defective in maintenance of CG methylation, *met1-3*, and non-CG methylation, *ddc*, are highly resistant to *Pseudomonas syringae* pv. *tomato* (*Pst*) infection (Dowen et al., 2012). *INCREASE IN BONSAI METHYLATION 1* (*IBM1*) negatively regulates CHG methylation in genic regions and mediates multiple developmental phenotypes, including flower and seed development (Saze et al., 2008). Mutants of *IBM1* demonstrate ectopic accumulation of H3K9me and CHG methylation, which are suppressed by mutation of *KYP/SUVH4* or *CMT3* (Saze et al., 2008). Genome-wide analysis of DNA methylation revealed that thousands of genes were hyper-methylated at CHG sites in *ibm1* mutants (Miura et al., 2009). Unlike DDM1, which controls CHG methylation at transposable element, pseudogenes, and repeat elements, *ibm1* mutation mainly affects long transcribed genes (Miura et al., 2009). IBM1 encodes a jumonji C (jmjC) domain, conserved for histone demethylase activity. JmjC demethylases preferentially remove monomethylated and dimethylated histone lysines (Inagaki et al., 2010), through an oxidative reaction that requires ferrous ion [Fe(II)] and α-ketoglutate as cofactors (Tsukada et al., 2006). Altogether, there are 21 annotated jmjC domain-containing protein in *Arabidopsis thaliana* and their roles in plant immunity is largely untouched. For instance, a few orphan studies recently demonstrated that *JMJ704* and *JMJ705* regulate defense in rice (Li et al., 2013; Hou et al., 2015).

Here, we report that IBM1 positively regulates *Arabidopsis* defenses against the hemi-biotrophic pathogen *Pst* DC3000. Loss of IBM1 repressed defense genes induction upon bacteria infection and PAMP perception. At the chromatic level, the reduced gene expression was associated with repressive H3 modifications. In addition, IBM1 directly associated with the gene body of *PR1*, *PR2*, and *FRK1* defense genes. We also explored the role of IBM1 in other defense pathways, including systemic acquired resistance, PTI, and defense against the necrotrophic pathogen, *Botrytis cinerea*. Overall, we revealed a novel role for IBM1 to maintain a permissive chromatin environment to ensure proper induction of defense genes under biotic stress.

## Materials and Methods

### Plant and Pathogen Materials

*Arabidopsis thaliana* ecotype Col-0 and the mutants, *ibm1-3* (SALK_023533) and *ibm1-4* (SALK_035608), were obtained from the Arabidopsis Biological Resource Center (http://abrc.osu.edu/). Seeds were surface sterilized in 10% bleach, washed with sterilized water, and kept for 3 days at 4°C. The sterilized seeds were then dispersed on 1/2 Murashige and Skoog (MS) medium containing 1% agar and grown for 14 days, under photosynthetic illumination (100 µE m^−2^ s^−1^) and short day condition (9-h-light, 22°C/15-h-dark, 18°C). Alternatively, seeds were stratified for 3 days, sown on commercial potting soil/perlite (3:2), and grown for 5 weeks, under the same growth conditions.

*P. syringae* pv. *tomato* (*Pst*) DC3000 and avirulent *Pst* DC3000 (*avrRpt2*) bacteria were obtained from B.N. Kunkel (Washington University, St. Louis, Missouri, USA). *Pst* DC3000 bacteria were grown at 28°C in King’s B medium supplemented with 50 mg/L rifampicin (Yekondi et al., 2017), and supplemented with 50 mg/L rifampicin and 50 mg/L kanamycin for *Pst* DC3000 (*avrRpt2*) bacteria.

The fungus *B. cinerea* (B071) was kindly provided by C.Y. Chen (National Taiwan University, Taipei, Taiwan). *B. cinerea* was grown at room temperature on potato dextrose agar (PDB)-agar plates as previously described (Zimmerli et al., 2001; Yekondi et al., 2017).

### Pathogen Infection Assays

For surface inoculation, 5-week-old plants were dip-inoculated with 10^6^ cfu/ml *Pst* DC3000 bacteria for 15 min and kept at 100% relative humidity for one night. Bacterial titers were quantified 3 days later on Kirby-Bauer (KB) agar plates as described previously (Huang et al., 2013). For infiltration inoculation, three fully expanded leaves of 5-week-old plants were infiltrated on the abaxial surface with 10^5^ cfu/ml *Pst* DC3000 bacteria using a needleless syringe. Bacterial titers were quantified on KB agar plates as described (Huang et al., 2013), after 3 days. For the systemic acquired resistance assay, three fully expanded leaves of 5-week-old plants were first infiltrated with 10^7^ cfu/ml *Pst* DC3000 (*avrRpt2*). Three other leaves were infiltrated 3 days later with 10^5^ cfu/ml *Pst* DC3000. Bacterial titers were quantified on KB agar plates as described (Huang et al., 2013), after 3 days. *B. cinerea* spores were diluted to 10^5^ spores/ml in 1/2 PDB medium and 10 µl droplets were deposited on leaf surface of 5-week-old plants (three leaves per plant). Leaves of the same age were chosen for droplet-inoculation. Plants were then kept at 100% relative humidity and lesion perimeters were determined after 3 days (Catinot et al., 2015).

### Gene Expression

For gene expression studies, 14-day-old seedlings were transferred to liquid 1/2 MS one night before treatment. *Pst* DC3000 bacteria were then added to reach a final concentration of 10^6^ cfu/ml for 3 h. Equivalent volume of 10 mM MgSO_4_ was used as mock control. Alternatively, flg22 was added to a final concentration of 100 nM for 3 h. Equivalent volume of water was used as mock control. For *B. cinerea* inoculation, spores were added to reach a final concentration of 10^5^ spore/ml for 24 h. Equivalent volume of 1/2 PDB medium was used as mock control. Samples were harvested and rinsed quickly in 1/2 MS, blotted dry, and snap frozen in liquid nitrogen. RNA was extracted with TRIzol reagent according to manufacturer’s instruction. First strand complementary DNA (cDNA) was synthesized with oligo dT and SuperScript III reverse transcriptase (Invitrogen). Quantitative PCR was performed using 2x SYBR green (Bio-Rad) and CFX96 real-time PCR system according to manufacturer’s instruction. Primers are listed in **Table S1** and **S2**.

### Chromatin Immunoprecipitation Assays

Chromatin immunoprecipitation (ChIP) assays were performed according to Lau and Bergmann (2015) with modifications. Briefly, 3 g of 14-day-old seedlings were harvested in 37 ml of cross-linking buffer [0.4 M sucrose, 10 mM Tris-HCl (pH 8), 10 mM MgCl2, and 1% (wt/vol) formaldehyde], followed by two rounds of vacuum infiltration, each for 5 min; 2.5 ml of 2 M glycine was then added, followed by vacuum infiltration for 5 min. Samples were rinsed with water, blotted dry, and grinded to fine powder in liquid nitrogen. The powder samples were resuspended in 40 ml of extraction buffer 1 [0.4 M sucrose, 10 mM Tris-HCl (pH 8), 10 mM MgCl2, and 5 mM β-mercaptoethanol] and incubated for 10 min before filtration through two layers of Miracloth (Millipore). The filtrates were centrifuged for 20 min at 3,000 g at 4°C using a swing-bucket rotor. The pellets were then resuspended in 1.3 ml of pre-chilled extraction buffer 2 [0.25 M sucrose, 10 mM Tris-HCl (pH 8), 10 mM MgCl2, 1% (vol/vol) Triton X-100, and 5 mM β-mercaptoethanol] and centrifuged for 10 min at 12,000 g at 4°C. The pellets were then resuspended in 400 µl of pre-chilled extraction buffer 3 [1.7 M sucrose, 10 mM Tris-HCl (pH 8), 2 mM MgCl2, 0.15% (vol/vol) Triton X-100, and 5 mM β-mercaptoethanol] and overlaid on another 400 µl of pre-chilled extraction buffer 3 in new tubes. Samples were centrifuged for 1 h at 16,000 g at 4°C. The pellets were resuspended in 500 µl of nuclei lysis buffer [50 mM Tris-HCl (pH 8), 10 mM EDTA, and 1% (wt/vol) SDS] and incubated on ice for 10 min. Finally, samples were sonicated with a 15 sec “ON,” 59 sec “OFF” cycle (x 40 cycles) at 40% output (Misonix 3000), to yield chromatin fragments with 150 base pair average length. Equal volume of the sonicated chromatin solution was set aside as input control.

For characterization of chromatin modification state, the sonicated extract was diluted 10 times with pre-chilled ChIP dilution buffer [16.7 mM Tris-HCl (pH 8), 167 mM NaCl, 1.2 mM EDTA, and 1.1% (vol/vol) Triton X-100], and immunoprecipitated with 10 µg of anti-H3K9me2 (Abcam, ab1220) or 10 µg of anti-H3K4me3 (Millipore, 07-473) antibody for 16 h At 4°C. Twenty microliters of pre-washed Magna ChIP Protein A+G Magnetic Beads (Millipore, 16-663) was then added to the chromatin-antibody mixture and incubated for 16 h At 4°C. For IBM1 targeting, the sonicated extract was diluted 10 times with pre-chilled ChIP dilution buffer, and immunoprecipitated with 20 µl of pre-washed GFP Trap-A beads (Chromotek) for 16 h At 4°C.

The magnetic beads were captured with a magnetic stand and washed successively with low-salt wash buffer [20 mM Tris-HCl (pH 8), 150 mM NaCl, 2 mM EDTA, 0.1% (wt/vol) SDS, and 1% (vol/vol) Triton X-100], high-salt wash buffer [20 mM Tris-HCl (pH 8), 500 mM NaCl, 2 mM EDTA, 0.1% (wt/vol) SDS, and 1% (vol/vol) Triton X-100], LiCl wash buffer [10 mM Tris-HCl (pH 8), 250 mM LiCl, 1 mM EDTA, 1% (vol/vol) NP-40, and 0.5% (wt/vol) sodium deoxycholate] and TE buffer [10 mM Tris-HCl (pH 8) and 1 mM EDTA]. Elution and reverse cross-linking was performed in a single step by adding 190 µl ChIP elution buffer [0.1 M NaHCO3 and 1% (wt/vol) SDS] and 8 µl of 5 M NaCl, to the input control and immunoprecipitated samples, followed by incubation at 65°C for 6 h. Chromatin DNA was purified by ChIP DNA Clean & Concentrator (Zymo Research) according to manufacturer’s instruction.

Quantification of chromatin DNA was performed with real-time quantitative PCR (qPCR) using specific primers listed in **Table S3**. Relative enrichment was represented by percentage of input, calculated by 2^−ΔCt^ (= 2^−[Ct(ChIP)−Ct(Input)]^) (Miura et al., 2009).

### Reactive Oxygen Species Burst

The reactive oxygen species (ROS) assay was carried out as described (Huang et al., 2013). Briefly, nine 0.25 cm^2^ leaf disks were excised from fully expanded leaves from 5-week-old *Arabidopsis* plants. The disks were incubated overnight in a 96-well plate with 100 µl of sterile water. Water was then replaced by 100 µl reaction solution [2 µl luminol (Sigma), 10 µg/ml horseradish peroxidase (Sigma)], with 100 nM flg22 or water (mock). The plate was analyzed at the indicated intervals for a period of 30 min using a CentroLIApc LB 692 plate luminometer [Berthold Technologies, (Bad Wildbad, Germany)].

### Callose Deposition

Fourteen-day-old seedlings were transferred to 1/2 MS liquid medium one night before inoculation with 1 x 10^6^ cfu/ml *Pst* DC3000 bacteria for 6 h. Harvested samples were cleared overnight by incubation in 95% ethanol at room temperature and then washed three times with sterile water. Cleared samples were stained with 0.01% aniline blue in 0.15 M phosphate buffer, pH 9.5 for 24 h. Callose deposits were visualized under UV illumination using an Olympus BX51 microscope. Quantification of callose deposits was performed on the acquired digital images using ImageJ (https://imagej.nih.gov/ij/).

### Accession Numbers

*IBM1* (AT3G07610), *PR1* (AT2G14610), *PR2* (AT3G57260), *FRK1* (AT2G19190), *PDF1.2a* (AT5G44420).

## Results

### IBM1 Positively Regulates *Arabidopsis* Resistance to Hemi-Biotrophic Bacteria

**Figure 1:**
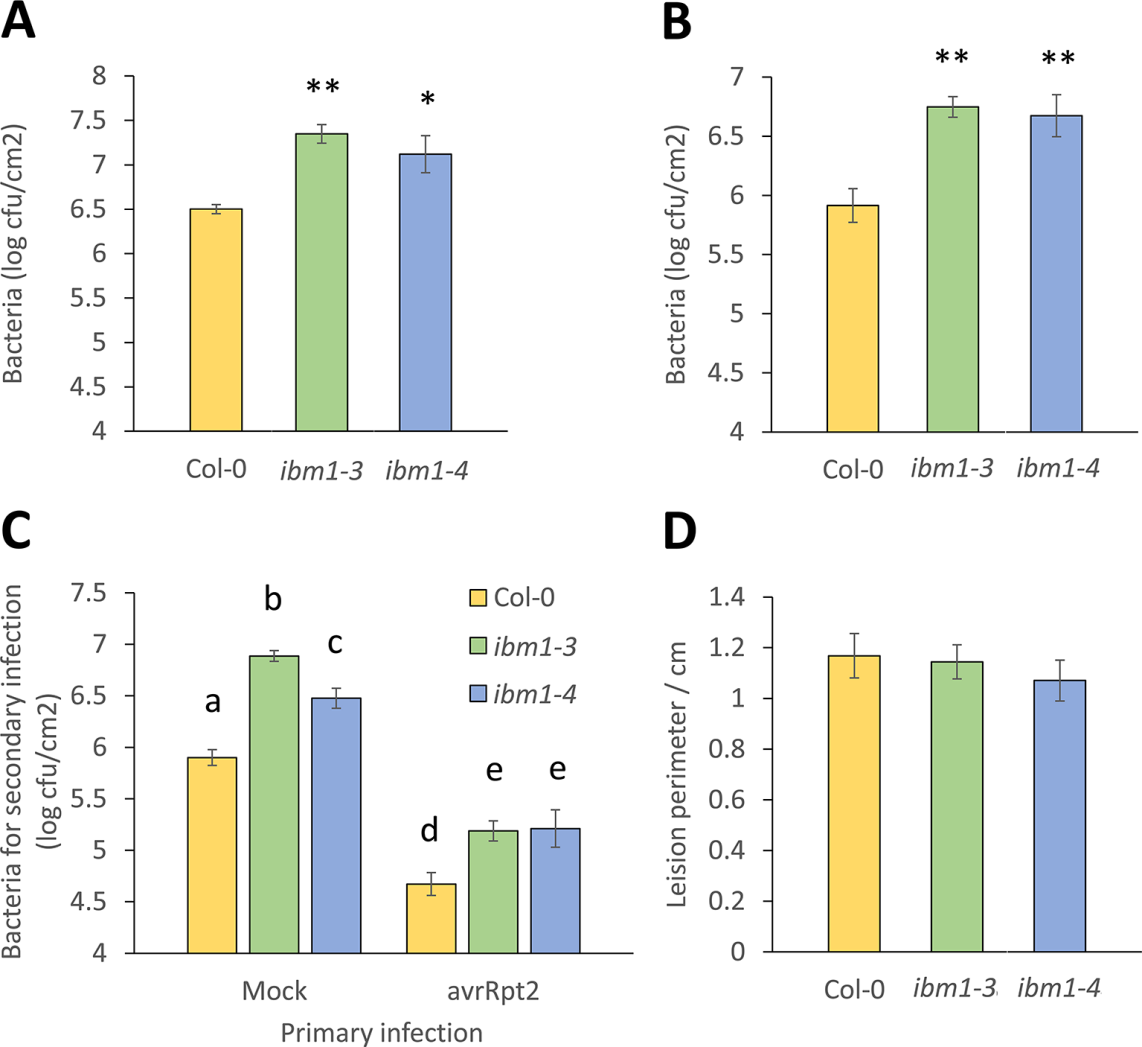
IBM1 positively regulates *Arabidopsis* resistance to *Pst* DC3000. **(A)** The *ibm1* mutants are hyper-susceptible to *Pst* DC3000. Five-week-old plants were dip-inoculated with 10^6^ cfu/ml *Pst* DC3000 for 15 min. Bacteria titers were evaluated at 3 dpi in Col-0, *ibm1-3*, and *ibm1-4*. Values represent average ± SEM from three independent experiments each with three plants (N = 9). Asterisks indicate significant differences from the Col-0 wild type (WT) as determined by a paired two-tailed Student’s *t*-test (*p < 0.05, **p < 0.01). **(B)** Susceptibility to primary *Pst* DC3000 infiltration inoculation. Three leaves of 5-week-old plants were infiltrated-inoculated with 10^5^ cfu/ml *Pst* DC3000. Bacteria titers were evaluated at 3 dpi in Col-0, *ibm1-3*, and *ibm1-4*. Values represent average ± SEM from three independent experiments each with at least three plants (N ≥ 9). Asterisks indicate significant differences from the Col-0 WT as determined by a paired two-tailed Student’s *t*-test (**p < 0.01). **(C)** Susceptibility to secondary *Pst* DC3000 infiltration inoculation. Three leaves of 5-week-old plants were first infiltrated-inoculated with 10^7^ cfu/ml of avirulent *Pst* DC3000 (*avrRpt2*), or with 10 mM MgSO_4_ as mock control. After 3 days, three other leaves were infiltrated-inoculated with 10^5^ cfu/ml *Pst* DC3000. Bacteria titers were evaluated at 3 dpi in Col-0, *ibm1-3*, and *ibm1-4*. Values represent average ± SEM from three independent experiments each with three plants (N = 9). Letters denote significant differences based on a one-way ANOVA with *post hoc* Tukey honestly significant difference (p < 0.05). **(D)** Susceptibility to *Botrytis cinerea*. Five-week-old plants were droplet-inoculated with *B. cinerea* (droplets of 10 μl with 10^5^ spores/ml) in 1/2 potato dextrose agar medium. Lesion perimeters were evaluated at 3 dpi in Col-0, *ibm1-3*, and *ibm1-4*. Values represent average ± SEM from three independent experiments each with at least three plants (N ≥ 9). No significant differences were observed as determined by a paired two-tailed Student’s *t*-test (p < 0.05).

In a screen to evaluate whether epigenetic regulators such as IBM1, met1, cmt3, drm1, drm2, drm3, ddm1, and hac1 are involved in *Arabidopsis* immunity to bacteria, mutants were dip-inoculated with virulent, hemi-biotrophic bacteria *Pst* DC3000 and disease symptoms were compared to respective wild-type (WT). From this screen, *ibm1* mutant plants were found to develop stronger disease symptoms. *IBM1* (At3g07610) encodes a histone H3K9 demethylase with a C-terminal jmjC domain known for histone demethylase activity. In the *ibm1-3* and *ibm1-4* mutants, the T-DNA is inserted in the sixth and ninth exon, respectively 827 and 2,173 base pairs downstream of the ATG start site (**Figure S1A**). Amplification of the genomic DNA and cDNA confirmed that *ibm1-3* and *ibm1-4* are both homozygous knock-out mutants (**Figures S1B, C**). To confirm the role of *IBM1* in resistance to bacteria, *ibm1-3* and *ibm1-4* plants were dip-inoculated with *Pst* DC3000 and disease symptoms and bacterial titers were evaluated at respectively 5 and 3 day-post-inoculation (dpi). Both loss-of-function mutants showed higher bacterial titers (**Figure 1A**) and increased symptoms (**Figure S2**) when compared to the Col-0WT control (**Figure 1A**). Similarly, both *ibm1-3* and *ibm1-4* mutants demonstrated increased susceptibility after infiltration inoculation with *Pst* DC3000 (**Figure 1B**). Furthermore, 3 days after a primary infection with avirulent *Pst* DC3000 (*avrRpt2*), three distal leaves of each plant received a second challenge inoculation with virulent *Pst* DC3000 and bacterial titers were determined 3 days later. As expected, bacterial titers in Col-0 WT preliminary treated with *Pst* DC3000 (*avrRpt2*) were significantly reduced when compared to Col-0 WT with mock primary infections (**Figure 1C**). Bacterial titers in the mutants were also significantly reduced in preliminary *Pst* DC3000 (*avrRpt2*)-inoculated plants, while bacteria counts were still significantly higher than the Col-0 WT controls (**Figure 1C**). This observation implies that IBM1 does not play a critical role in systemic acquired resistance. To further evaluate the role of IBM1 in resistance against deleterious pathogens, *ibm1-3* and *ibm1-4* plants were droplet-inoculated with *B. cinerea*, a necrotrophic fungal pathogen (Zimmerli et al., 2001). When lesion perimeters and symptoms were determined at 3 dpi, no significant differences were observed between *ibm1* mutants and the Col-0 WT (**Figure 1D** and **Figure S2**). Taken together, these data suggest that IBM1 is required for basal level of resistance against hemi-biotrophic bacteria but not for systemic acquired resistance, nor resistance against necrotrophic pathogens.

### Up-Regulation of Defense Marker Genes Is Compromised in *ibm1* Mutants

**Figure 2:**
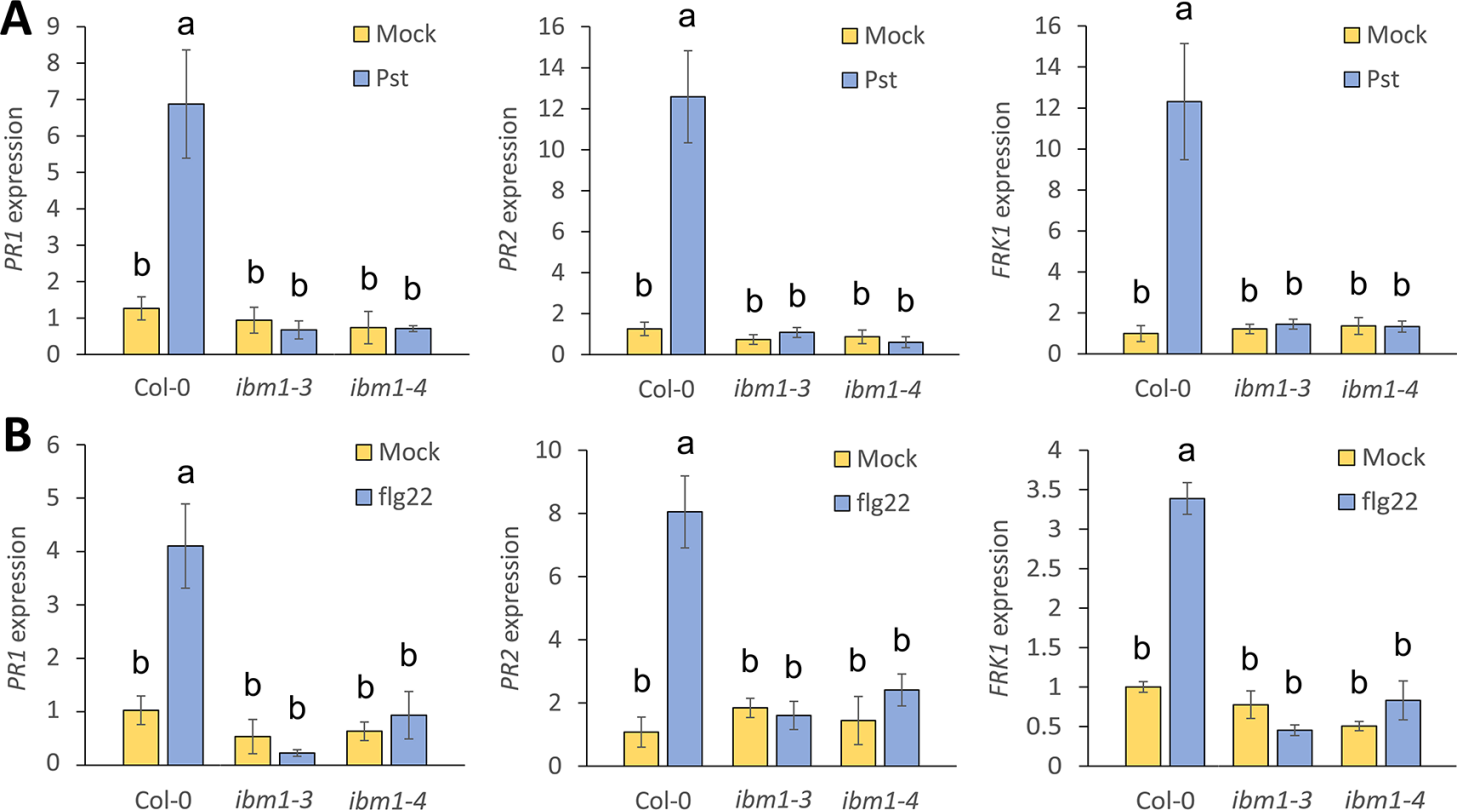
Up-regulation of immunity marker genes is compromised in *ibm1* mutants. **(A)** Up-regulation of defense marker genes after *Pst* DC3000 inoculation. Fourteen-day-old seedlings were floated in liquid 1/2 Murashige and Skoog (MS) for one night before inoculation with 10^6^ cfu/ml *Pst* DC3000 for 3 h. Equivalent volume of 10 mM MgSO_4_ was used as mock control. Transcript levels of *PR1*, *PR2*, and *FRK1* were determined by quantitative real-time PCR and normalized to *UBQ10* (Col-0 mock as defined value of 1). Values represent average ± SEM from three independent experiments each with three technical repeats (N = 9). Letters denote significant differences based on a one-way ANOVA with *post hoc* Tukey honestly significant difference (p < 0.05). **(B)** Up-regulation of defense marker genes after flg22 treatment. Fourteen-day-old seedlings were floated in liquid 1/2 MS for one night before treatment with 100 nM flg22 for 3 h. Equivalent volume of water was used as mock control. Gene expression of *PR1*, *PR2*, and *FRK1* were evaluated and analyzed as in A.

IBM1 controls flowering, seed and shoot development (Saze et al., 2008). However, the role of IBM1 in biotic stress is largely unknown. To test whether IBM1 is critical for the regulation of defense related genes, we analyzed the expression of *PR1*, *PR2*, and the PTI marker *FRK1* 3 h after inoculation with *Pst* DC3000. As expected, *PR1*, *PR2*, and *FRK1* were strongly up-regulated in Col-0 WT controls after *Pst* DC3000 inoculation (**Figure 2A**). By contrast, up-regulation of *PR1*, *PR2*, and *FRK1* was abolished in *ibm1* mutants (**Figure 2A**). These data are consistent with the observed hyper-susceptibility phenotype of *ibm1* mutants (**Figures 1A, B**). Similarly, treatment with flg22, a 22-amino acid peptide derived from the terminus of the PAMP flagellin (Gomez-Gomez and Boller, 2000), induced *PR1*, *PR2*, and *FRK1* expression in Col-0 WT but not in the *ibm1* mutants (**Figure 2B**). On the other hand, upon inoculation with *B. cinerea* spores, the expression of *PDF1.2a*, a known marker gene for necrotrophic attack (Thomma et al., 1998; Zimmerli et al., 2001), was not affected in the *ibm1* mutants (**Figure S3**). Notably, significant up-regulation of *IBM1* gene expression was not observed upon *Pst* DC3000 inoculation or after flg22 treatment (**Figure S4**). Together these data suggest that IBM1 is required for basal defense response activation without being induced by pathogen elicitation.

### IBM1 Controls the Chromatin Modification States At *PR1*, *PR2*, and *FRK1* Loci

**Figure 3:**
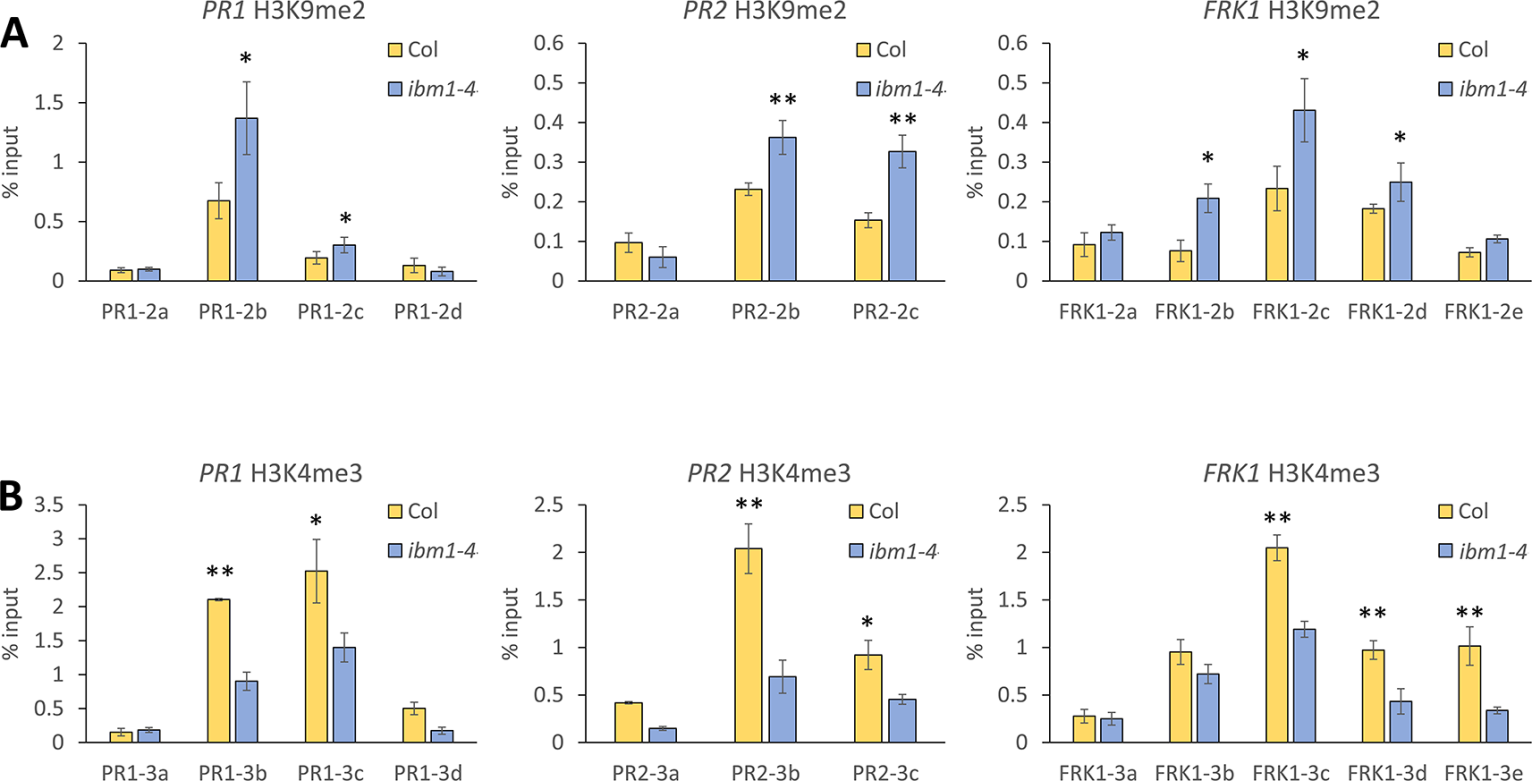
IBM1 modulates the chromatin state of defense marker genes. **(A)** Detection of H3K9me2 levels. Tissue of 14-day-old seedlings were pooled and chromatin immunoprecipitation was carried out using anti-H3K9me2 antibody for Col-0 and *ibm1-4*. The associated chromatin was quantified by quantitative PCR with primers spanning across *PR1*, *PR2*, and *FRK1*. Relative enrichments were calculated as percentage of input. Values represent average ± SD from four technical repeats (N = 4). The experiment was repeated twice with similar patterns and one representative repeat is shown. Asterisks indicate significant differences from respective Col-0 wild type controls as determined by a paired two-tailed Student’s *t*-test (*p < 0.05, **p < 0.01). **(B)** Detection of H3K4me3 levels for *PR1*, *PR2*, and *FRK1* were evaluated and analyzed as in A with anti-H3K4me3 antibody.

IBM1 preferentially demethylates H3K9 at low-copy loci to protect transcribed genes from DNA methylation at CHG sites (Miura et al., 2009). To address whether IBM1 regulates the expression of *PR1*, *PR2*, and *FRK1* by modulating histone methylation of these loci, we applied the ChIP assay followed by qPCR quantification and used a panel of primers spanning across these defense-related loci (**Figure S5**). Higher levels of H3K9me2, an inactivation mark, in the *ibm1-4* mutant for *PR1*, *PR2*, and *FRK1* were observed (**Figure 3A**). In addition, the levels of H3K4me3, an activation mark (Fan et al., 2012), were significantly reduced in the *ibm1* mutant for *PR1*, *PR2*, and *FRK1* (**Figure 3B**). Together, these data are consistent with the observed defective up-regulation of these defense genes upon bacterial attack. On the other hand, *PDF1.2a* showed no significant difference for both H3K9me2 and H3K4me3 levels (**Figure S6**). Therefore, IBM1 is required for the suppression of the repressive histone mark H3K9me2 and the accumulation of the activation histone mark H3K4me3 at *PR1*, *PR2*, and *FRK1*.

### IBM1 Associates Directly With *PR1*, *PR2*, and *FRK1* Chromatin

**Figure 4:**
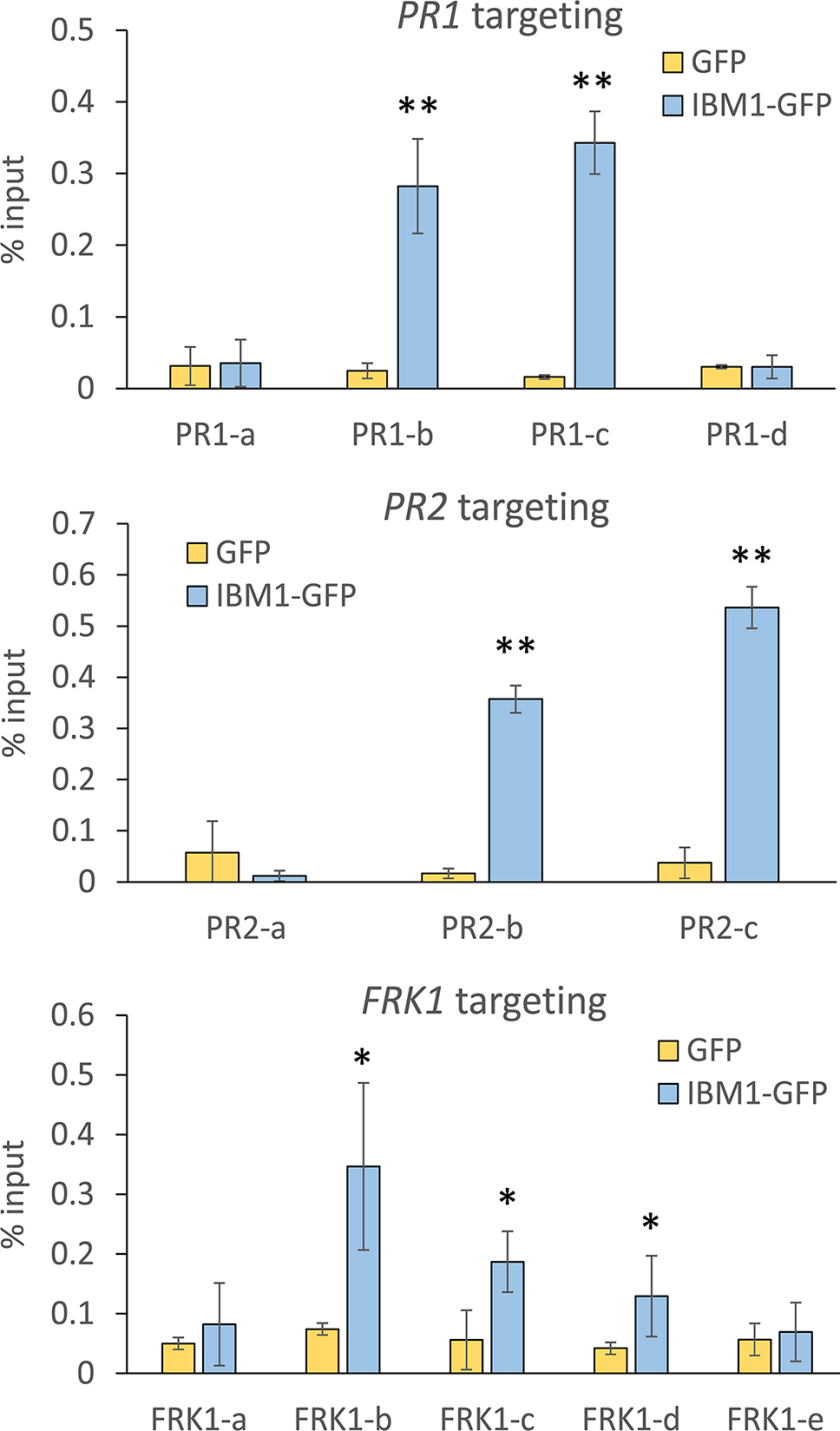
IBM1 directly associates with the chromatin of defense genes. Tissues of 14-day-old seedlings were pooled and chromatin immunoprecipitation was carried out using anti-green fluorescent protein (GFP) magnetic beads for *ProIBM1::IBM1-GFP* in the *ibm1-4* mutant background (IBM1-GFP) and GFP transgenic plants. The associated chromatin was quantified by quantitative PCR with primers spanning across *PR1*, *PR2*, and *FRK1*. Relative enrichment was calculated as percentage of input. Values represent average ± SD from four technical repeats (N = 4). The experiment was repeated twice with similar patterns and one representative repeat is shown. Asterisks indicate significant differences from respective GFP controls as determined by a paired two-tailed Student’s *t*-test (*p < 0.05, **p < 0.01).

To investigate whether IBM1 directly associates with the chromatin of *PR1*, *PR2*, and *FRK1*, ChIP assays were used with transgenic plants expressing *ProIBM1::IBM1-GFP* in the *ibm1-4* mutant background. Chromatin associated with IBM1 was immunoprecipitated using anti-green fluorescent protein (GFP) magnetic beads and quantified by qPCR. Significant enrichments of *PR1*, *PR2*, and *FRK1* DNA fragments in IBM1-GFP transgenic plants were observed when compared to GFP only controls (**Figure 4**). On the other hand, significant enrichments of *PDF1.2a* DNA fragments in IBM1-GFP transgenic plants were not observed (**Figure S7**). Taken together, our data suggest that IBM1 associates directly with the gene body of *PR1*, *PR2*, and *FRK1*. Thus, IBM1 may modulate the chromatin modification state of these loci. Notably, *PR1*, *PR2*, and *FRK1* are hyper-methylated in *ibm1* mutants and hence up-regulation upon bacterial infection is hindered, leading to hyper-susceptibility to *Pst* bacteria.

### IBM1 Does Not Play an Important Role in Apoplastic Pattern-Triggered Immunity

**Figure 5:**
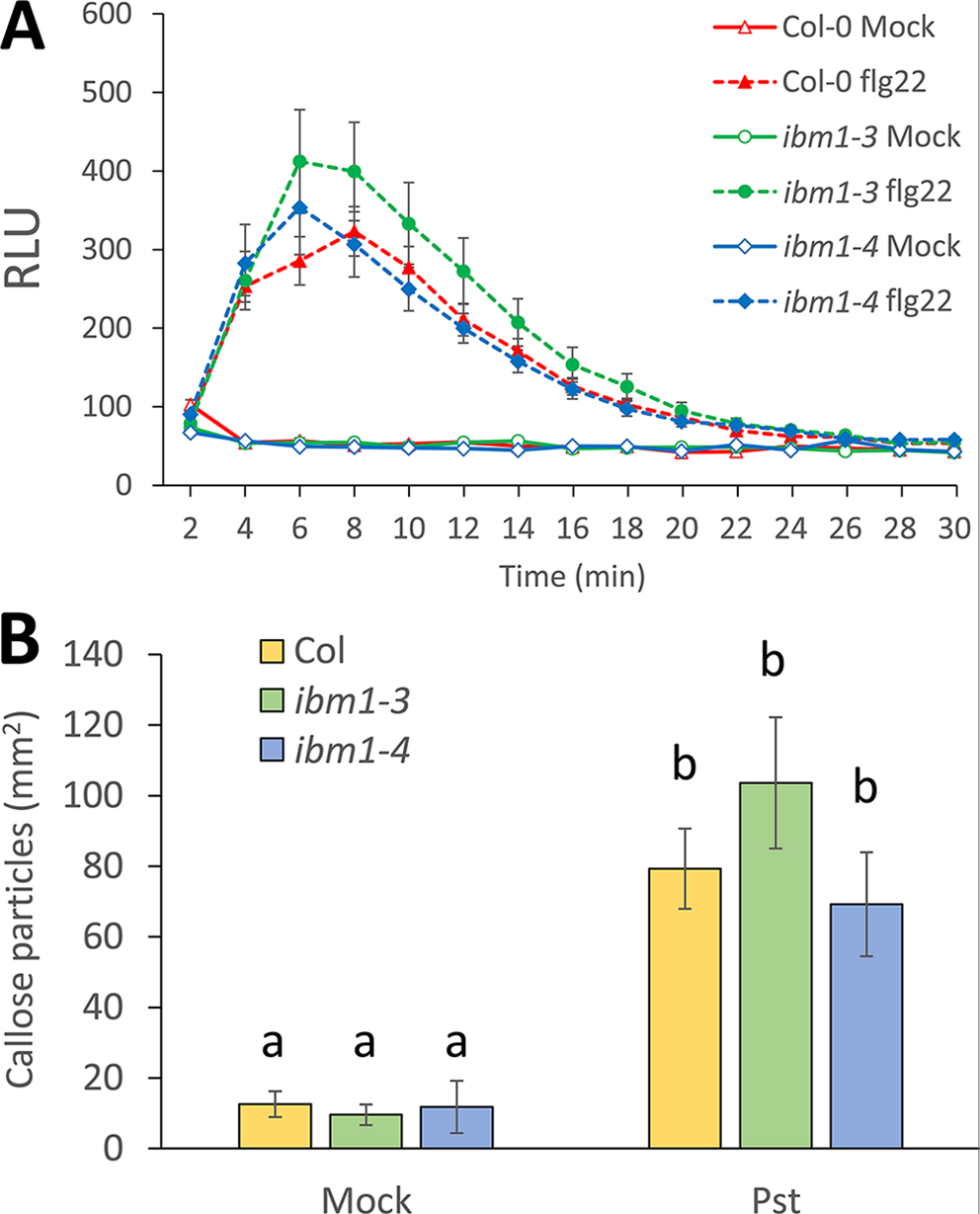
The role of IBM1 in apoplastic PTI. **(A)** Reactive oxygen species production. Leaf disks from 5-week-old plants were treated with 100 nM flg22 or water (mock). Relative light units (RLUs) were evaluated at the indicated time points. Values represent average ± SEM from three independent experiments, each consisting of six leaf disks (N = 18). No significant differences to the Col-0 wild type (WT) were observed when analyzed with a paired two-tailed Student’s *t*-test (p < 0.05). **(B)** Callose deposition. Fourteen-day-old seedlings were floated in liquid 1/2 Murashige and Skoog for one night before treatment with 10^6^ cfu/ml *Pst* DC3000 for 6 h. Equivalent volumes of 10 mM MgSO_4_ were used for mock controls. Values represent average ± SEM from three independent experiments, each consisting of at least three seedlings (N = 9). No significant differences to respective Col-0 WT were observed when analyzed with a one-way ANOVA with *post hoc* Tukey honestly significant difference (p < 0.05).

Since *FRK1* is an important marker for PTI (Gomez-Gomez et al., 1999; Xiao et al., 2007), we also investigated the potential role of IBM1 in other apoplastic PTI responses. As an early PTI response, we first analyzed the production of ROS after treatment with 100 nM flg22. No significant differences between the Col-0 WT, *ibm1-3*, and *ibm1-4* were observed (**Figure 5A**). Pathogen- or PAMP-mediated callose deposition is considered an important late PTI response (Zipfel and Robatzek, 2010; Yeh et al., 2016). Callose deposition was thus evaluated after inoculation with *Pst* DC3000 bacteria. Aniline blue staining and image analysis revealed that the increase in callose deposition in the Col-0 WT control, *ibm1-3*, and *ibm1-4* mutants were at similar levels (**Figure 5B**). Taken together, our data suggest that IBM1 mainly plays a role in regulating defense gene expression and is not critical in other apoplastic PTI responses.

### Repression of *ibm1*-Induced Immunity Defects

*Ibm1* mutants display a number of developmental defects due to ectopic H3K9me2 and/or CHG methylation in genic regions (Rigal et al., 2012). Mutations in *kyp* and *ldl2* suppress *ibm1* mutants developmental abnormalities (Rigal et al., 2012). To address whether *ibm1*-induced immunity defect can also be rescued by mutations in *kyp* and *ldl2*, *kypibm1*, and *ldl2ibm1* double mutants were challenged with *Pst* DC3000. After infiltration inoculation, *kypibm1* and *ldl2ibm1* harbored bacterial titers similar to *ibm1-4* (**Figure S8**), indicating that these double mutants are hyper-susceptible to *Pst* DC3000. These data suggest that in contrary to developmental defects, mutations in *kyp* and *ldl2* cannot suppress *ibm1* defective immunity to *Pst* DC3000.

## Discussion

In this work, we provide physiological and molecular evidences to show that the epigenetic regulator IBM1 plays a key role in maintaining *Arabidopsis* basal immunity to bacteria. More specifically, the role of IBM1 on the expression of defense genes upon bacteria infection and PAMP perception is highlighted.

### IBM1 Positively Regulates *Arabidopsis* Resistance to *Pst* DC3000 Bacteria

Loss of IBM1 increased plant susceptibility to the bacterial pathogen *Pst* DC3000. In addition, upon bacteria inoculation or treatment with the PAMP flg22, *ibm1* mutants failed to show up-regulation of a subset of defense genes, including *PR1*, *PR2*, and *FRK1*. DNA methylation at CG or non-CG sites, and histone modification at H3K4, H3K9, H3K27, and H3K36 have been associated with plant defense (Berr et al., 2010; Dowen et al., 2012; Li et al., 2013; Singh et al., 2014). For example, mutants defective in DNA methylation such as *met1-3* and *ddc* are highly resistant to bacteria, and harbor a mis-regulated *Pst*-induced transcriptional regulatory network (Dowen et al., 2012). Notably, *PR1* expression levels are significantly higher in both *met1-3* and *ddc* mutants than in WT controls after *Pst* infection (Dowen et al., 2012). The *PR1* locus is not a direct target for DNA methylation, so the observed altered expression is believed to be an indirect consequence of other epigenetic modification further upstream (Dowen et al., 2012). In this study however, the observed reduced H3K4me3 and increased H3K9me2 in *ibm1-4* mutant may be the primary cause for the failed up-regulation of defense genes and increased susceptibility.

*SDG8*, a SET DOMAIN GROUP8 methyltransferase mediates H3K38 dependent defense gene expression against necrotrophic fungal pathogens (Berr et al., 2010). To test whether IBM1 also plays a role in *Arabidopsis* immunity against fungal pathogens, *ibm1* mutants were challenged with the necrotrophic fungal pathogen *B. cinerea*. By contrast to infection by *Pst* DC3000 bacteria, *ibm1* mutants showed WT resistance to *B. cinerea*. Consistently, the up-regulation of *PDF1.2a*, a critical defense gene activated upon infection with necrotrophs (Berr et al., 2010), was not affected in the *ibm1* mutants after inoculation with *B. cinerea* spores. Therefore, IBM1 may only play a role in regulating resistance and defense gene expression under bacterial infection.

### IBM1 Is Not Critical for Apoplastic Pattern-Triggered Immunity Responses

Apoplastic PTI is a complex set of responses crucial for resisting pathogen attack (Boller and Felix, 2009). The first line of defense involves the recognition of PAMPs by cell surface pattern recognition receptors (PRRs) such as FLS2 and EF-TU Receptor (EFR) that recognize the conserved N-terminus of bacteria flagellin (Gomez-Gomez and Boller, 2000), and the bacterial protein elongation factor-Tu (Kunze et al., 2004), respectively. Other players, including malectin-like/leucine rich repeat receptor-like kinases (Yeh et al., 2016; Stegmann et al., 2017), lectin receptor kinases (Bouwmeester and Govers, 2009; Bouwmeester et al., 2011; Desclos-Theveniau et al., 2012; Singh et al., 2012; Huang et al., 2014) and cysteine-rich receptor-like kinases (Bourdais et al., 2015; Yeh et al., 2015), among others, act as agonists or antagonists of the PRR complexes to fine tune PTI activation and silencing. Recognition of PAMPs by PRRs is usually followed by the accumulation of ROS and callose (Zipfel and Robatzek, 2010). So far, the characterization of epigenetic regulators in relation to plant immunity mainly focuses on the correlation between the disease phenotype, defense gene expression, and DNA methylation/histone modification patterns (Espinas et al., 2016; Li et al., 2016). Investigation of other aspects of the PTI response is largely deficient. Yet, HAC1 was reported to modulate some aspects of PTI, including deposition of callose (Singh et al., 2014). In this report, ROS burst and callose deposition were selected as PTI outcomes not linked to gene expression to further investigate the role of IBM1 in PTI. No significant differences between *ibm1* mutants and Col-0 WT in ROS accumulation and callose deposition upon bacterial inoculation and after PAMP treatment were observed. These data suggest that IBM1 mainly controls histone methylation patterns at selected loci, and hence the expression of specific defense genes, but is likely not a regulator of other immunity responses.

### IBM1 as a Chromatin Modification Regulator in Plant-Microbe Interaction

Depending on their specific target loci, posttranslational histone modifications and DNA methylation can play both positive and negative roles in regulating gene transcription (Zhu, 2009). Notably, *SDG27* positively regulates H3K4me3 patterns at the key defense related locus *WRKY70*, but not at *PR1* nor at *THI2.1* (Alvarez-Venegas et al., 2006). Similarly, the *sdg8* mutant is hyper-susceptible to fungal pathogens due to a loss of H3K36me3-mediated activation of *PDF2.1*, *VSP2*, *ERF1*, and *MYC2* (Berr et al., 2010). By contrast, *JMJ705* promotes rice resistance against bacterial blight *via* reduction of H3K27me3 levels at *PR5* and *PR10* (Li et al., 2013). In this report, we show that IBM1 is required for *Arabidopsis* full resistance to *Pst* DC3000 infection. The defective defense response in *ibm1* mutants is correlated with a loss of up-regulation of defense marker genes such as *PR1*, *PR2*, and *FRK1*. In addition, *ibm1* mutants harbored an increased accumulation of the inactivation histone mark H3K9me2 and a reduction of the activation mark H3K4me3 at these defense loci. Unlike *SDG27*, which maintains H3K4me3 at the promoter of *WRKY70* (Alvarez-Venegas et al., 2007), IBM1 maintained H3K4me3 at the gene body of *PR1*, *PR2*, and *FRK1*. Although mutations in epigenetic regulators such as *kyp* and *ldl2* are known to suppress the epigenetic and developmental defect of *ibm1* mutants (Rigal et al., 2012), mutations in *kyp* and *ldl2* could not rescue the *ibm1*-induced immunity defect (**Figure S8**). KYP and LDL2 act on histone methylation at chromatin level (Saze et al., 2012; Du et al., 2014; Inagaki et al., 2017), and apparently the absence of functional KYP and LDL2 cannot restore full resistance in *ibm1*. Therefore, like *JMJ705* in rice (Li et al., 2013), IBM1 may be critical to *Arabidopsis* resistance to *Pst* DC3000 *via* a direct regulatory role at defense genes.

## Concluding Remarks

This work shows that IBM1 is required for *Arabidopsis* full resistance to *Pst* DC3000 infection. IBM1 did not play an important role in systemic acquired resistance, nor resistance to necrotrophic pathogens such as *B. cinerea*. The defective defense response in *ibm1* mutants may be primarily due to the loss of up-regulation of defense genes, including *PR1*, *PR2*, and *FRK1* upon bacterial infection. The accumulation of the inactivation histone mark H3K9me2 and the reduction of the activation mark H3K4me3 at these defense loci may explain the defective up-regulation of these defense genes. This study also demonstrated the direct association of IBM1 with the chromatin of *PR1*, *PR2*, and *FRK1*. In addition, analyses of ROS production and callose deposition suggest that IBM1 does not play an important role in other PTI responses. Taken together, our results highlight the importance of IBM1 in plant immunity through the control of defense genes *via* histone modification.

## Data Availability Statement

All datasets generated for this study are included in the article/**Supplementary Material**.

## Author Contributions

CC and LZ designed the study and wrote the manuscript. CC performed the experiments.

## Funding

This work was supported by the Ministry of Science and Technology of Taiwan, grants 102-2628-B-002-011-MY3 and 105-2311-B-002-032-MY3 (to LZ).

## Conflict of Interest

The authors declare that the research was conducted in the absence of any commercial or financial relationships that could be construed as a potential conflict of interest.
