# Supplementary material for: The Histone Demethylase IBM1 Positively Regulates Arabidopsis Immunity by Control of Defense Gene Expression
Source: Front Plant Sci. 2019 Dec 18;10:1587. doi: 10.3389/fpls.2019.01587 (PMC6951416; doi:10.3389/fpls.2019.01587)
Supplement: Supplementary file 1 [file DataSheet_1.docx]

**Supporting Information**


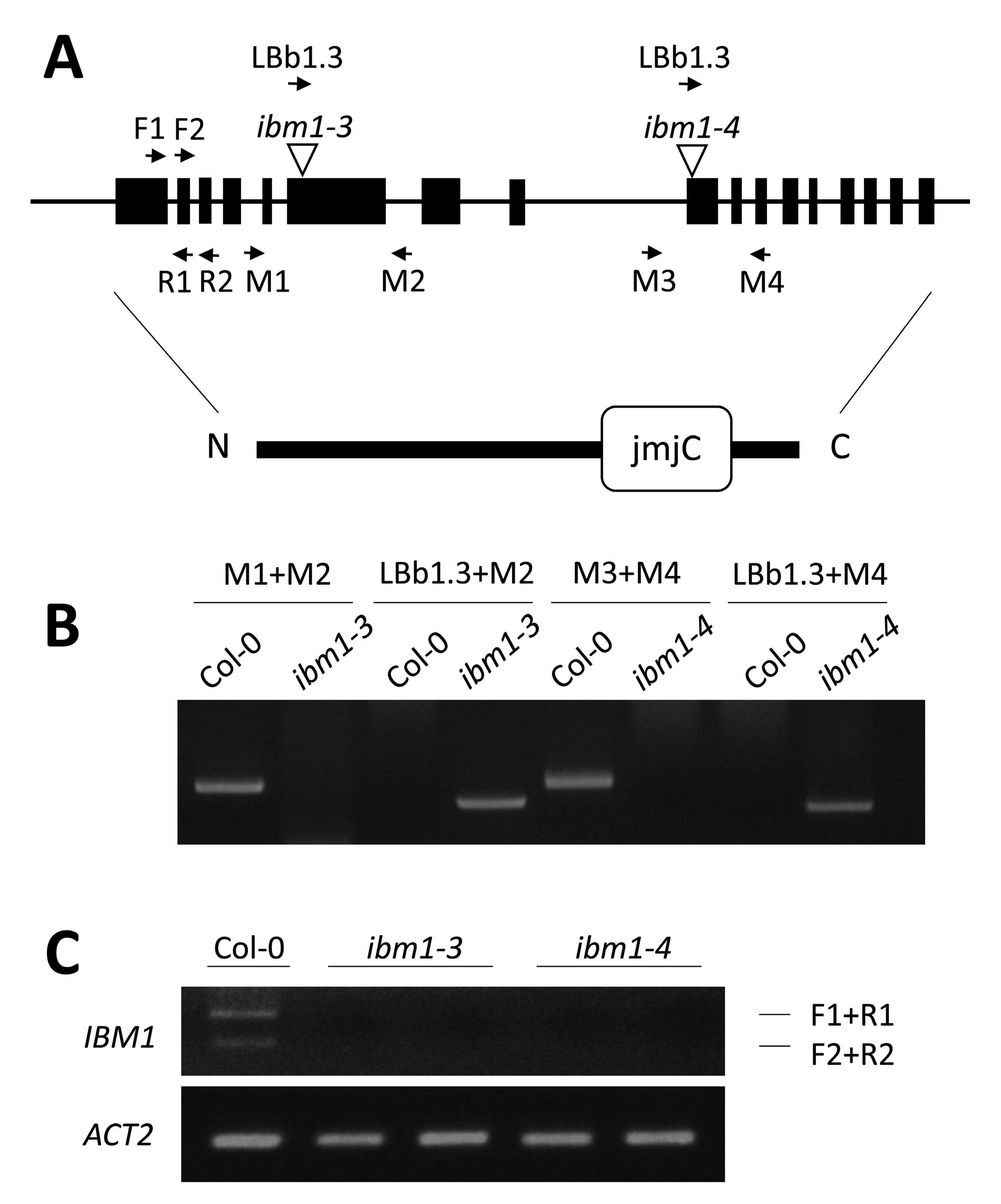


**Figure S1 |** Characterization of *ibm1-3* and *ibm1-4* mutants. **(A)** *IBM1* gene structure. The T-DNA insertion is in the sixth and ninth exon for *ibm1-3* and *ibm1-4* respectively. M1, M2 and LBb1.3 primers were used for the genotyping of *ibm1-3*. M3, M4 and LBb1.3 primers were used for the genotyping of *ibm1-4*. F1, R1, F2 and R2 primers were used for expression analysis. Primer sequences are listed in Table S1. IBM1 possesses a jmjC domain. **(B)** Genotyping of *ibm1* mutants. M1 + M2: wild-type allele, LBb1.3 + M2: *ibm1-3* mutant allele, M3 + M4: wild-type allele and LBb1.3 + M4: *ibm1-4* mutant allele. **(C)** *ibm1-3* and *ibm1-4* are knock-out mutants. RT-PCRs were performed with primers described in Fig 1A spanning exon 1 and 2 (F1+R1), exon 2 and 3 (F2+R2). *ACTIN2* (*ACT2*) was used as internal control.


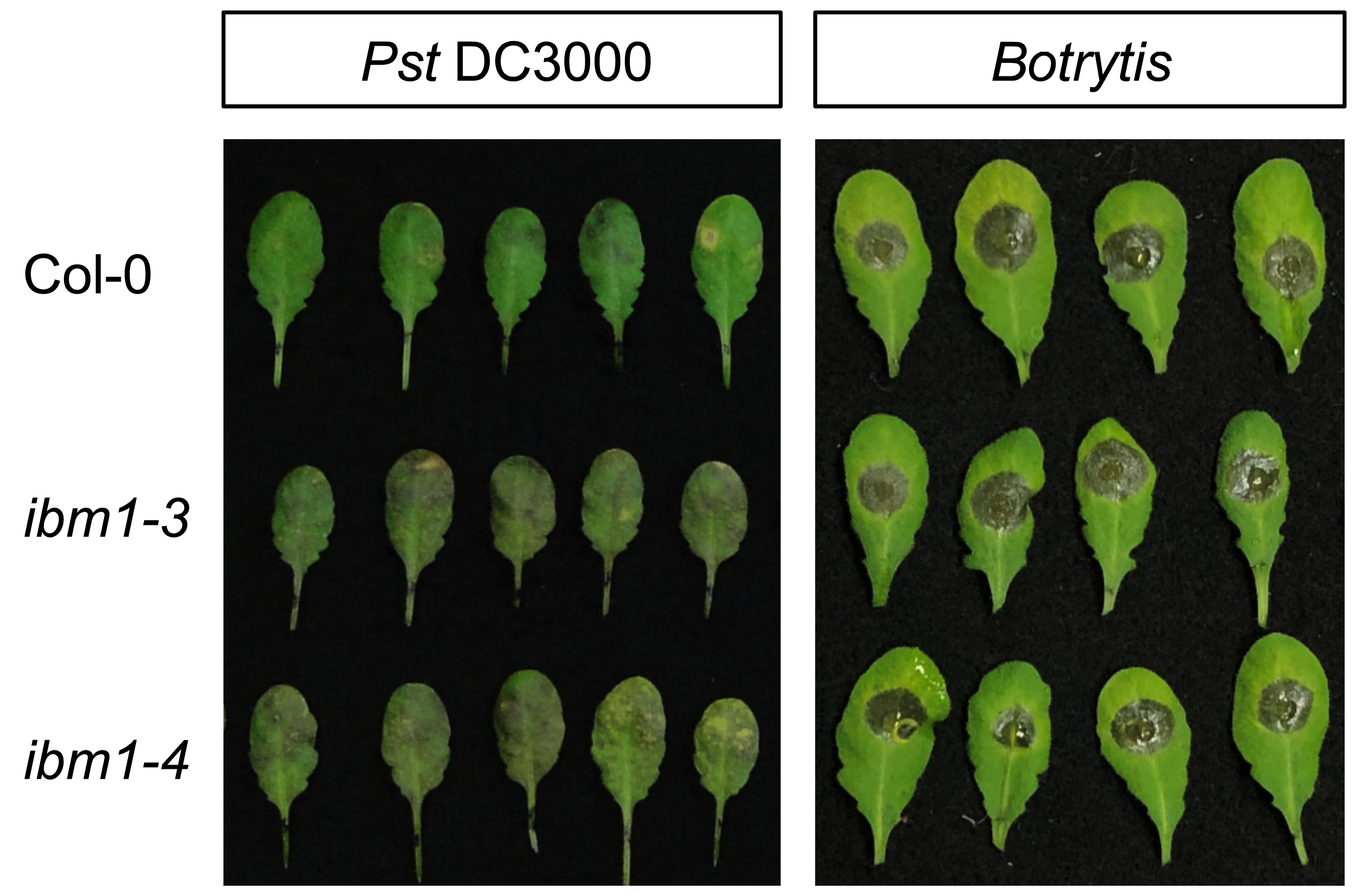


**Figure S2 |** Symptoms after pathogen infection in *ibm1* mutants. Five-week-old plants were dip-inoculated with 10^6^ cfu/mL *Pst* DC3000 for 15 min. Symptoms were photographed at 5 dpi in Col-0, *ibm1-3* and *ibm1-4*. For *B. cinerea* infection, five-week-old plants were droplet-inoculated with droplets of 10 μL with 10^5^ *B. cinerea* spores/mL in 1/2 PDB medium. Pictures were taken at 3 dpi. Experiments were repeated at least twice with similar results.

**
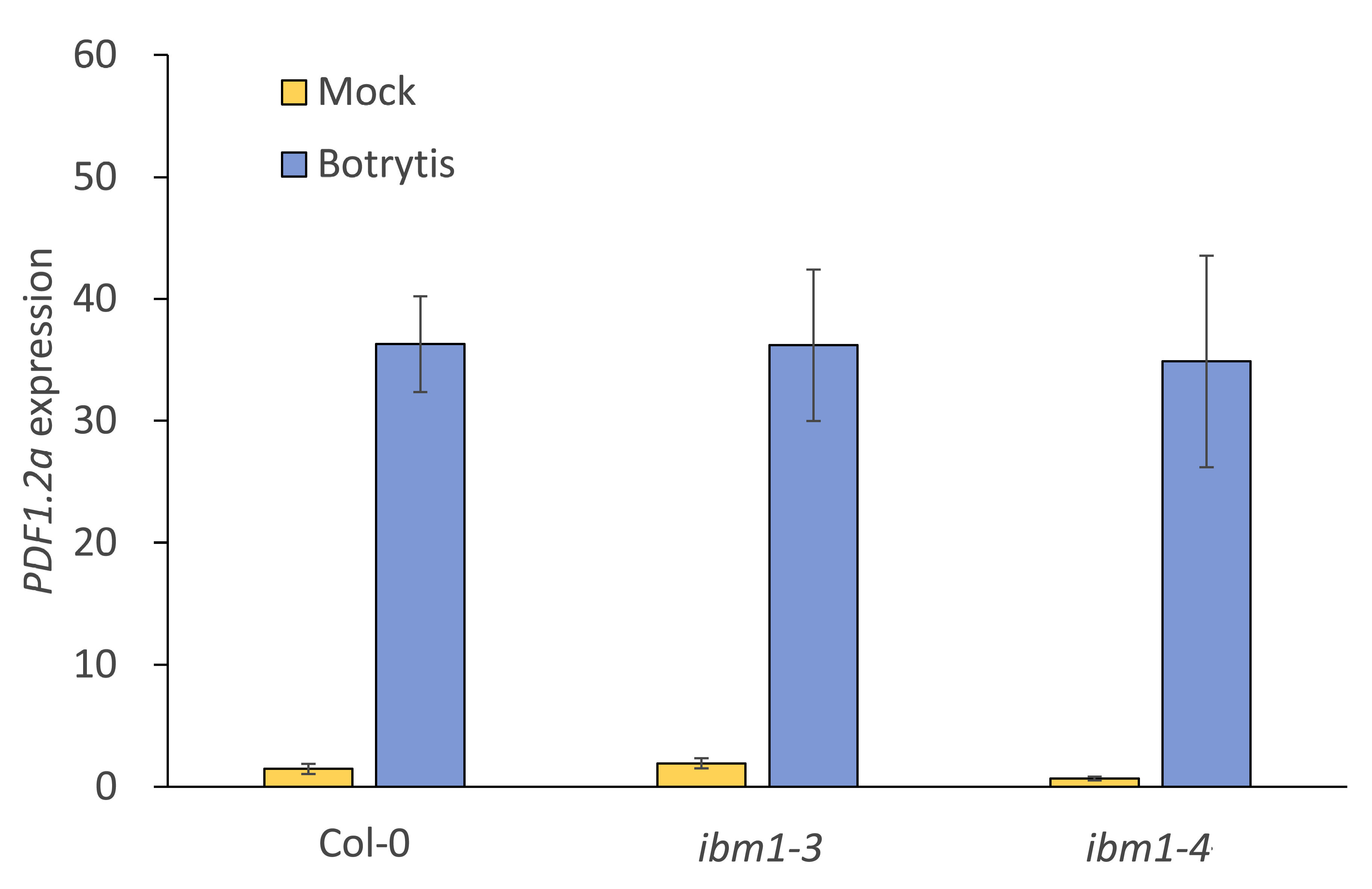
**

**Figure S3 |** Up-regulation of *PDF1.2a* is unaffected in *ibm1* mutants. Up-regulation of *PDF1.2a* after *B. cinerea* inoculation. Fourteen-day-old seedlings were floated in liquid ½ MS for one night before inoculation with *B. cinerea* (10^5^ spore/mL) for 24 h. Equivalent volume of ½ PDB was used as mock control. Transcript levels of *PDF1.2a* were determined by qRT-PCR and normalized to UBQ10 (Col-0 Mock as defined value of 1). Primer sequences are listed in Table S2. Values represent average ± SEM from 3 independent experiments each with 3 technical repeats (N = 9). No significant differences were observed from the Col-0 WT control as determined by a paired two-tailed Student’s *t*-test (p < 0.05).


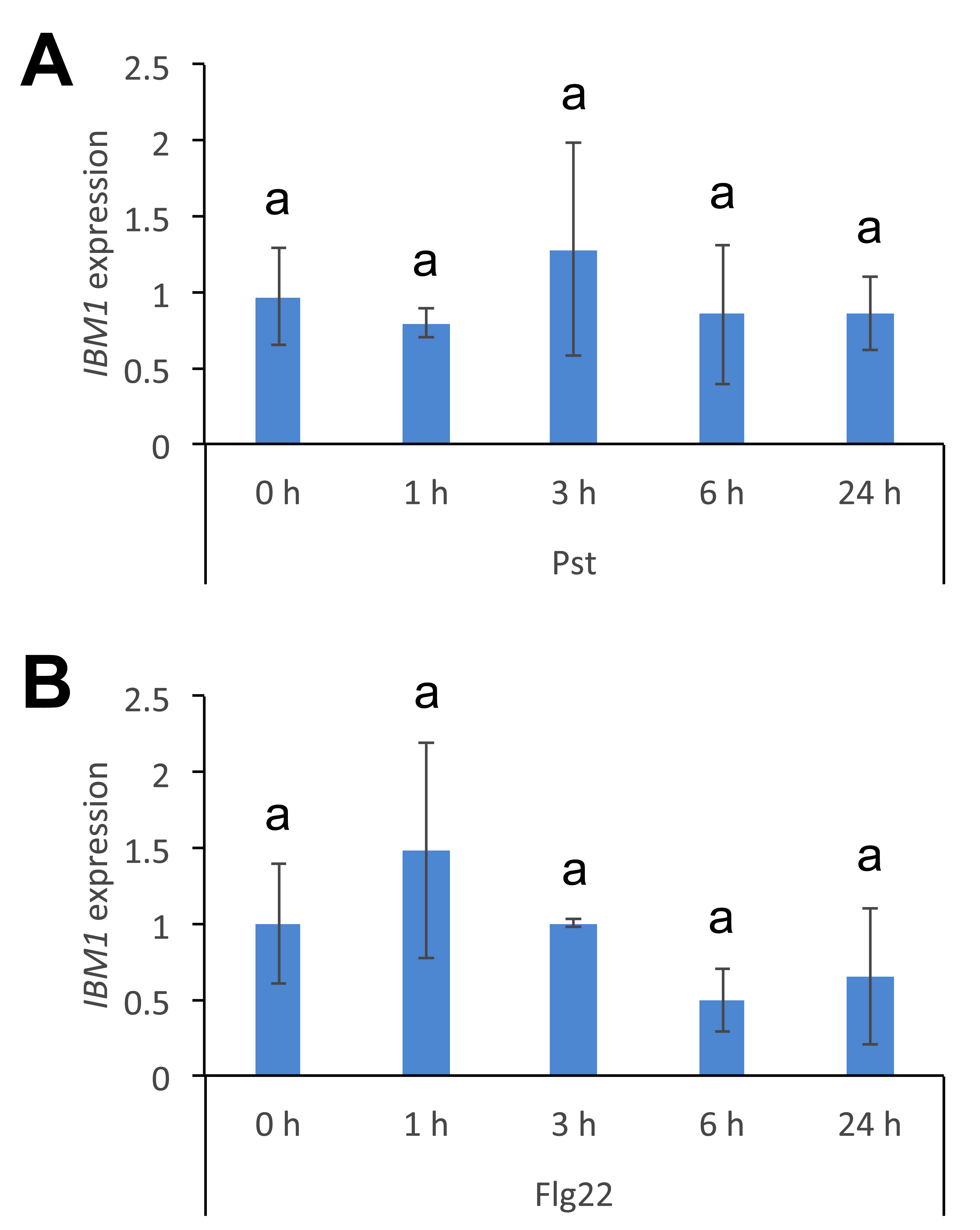


**Figure S4 |** Neither *Pst* inoculation nor flg22 treatment alters *IBM1* gene expression. **(A)** *IBM1* expression after *Pst* DC3000 inoculation. Fourteen-day-old Col-0 seedlings were floated in liquid 1/2 MS for one night before inoculation with 10^6^ cfu/mL *Pst* DC3000 and harvested at the indicated time points. Transcript levels of *IBM1* were determined by qRT-PCR and normalized to *UBQ10* (Col-0 Mock as defined value of 1). Values represent average ± SEM from 3 independent experiments each with 3 technical repeats (N = 9). No significant differences were observed from the control as determined by a one-way ANOVA with post-hoc Tukey HSD (p < 0.05). **(B)** *IBM1* expression after flg22 treatment. Fourteen-day-old seedlings were floated in liquid 1/2 MS for one night before treatment with 100 nM flg22 for 3 h. Gene expression of *IBM1* was evaluated and analyzed as in (A).

**
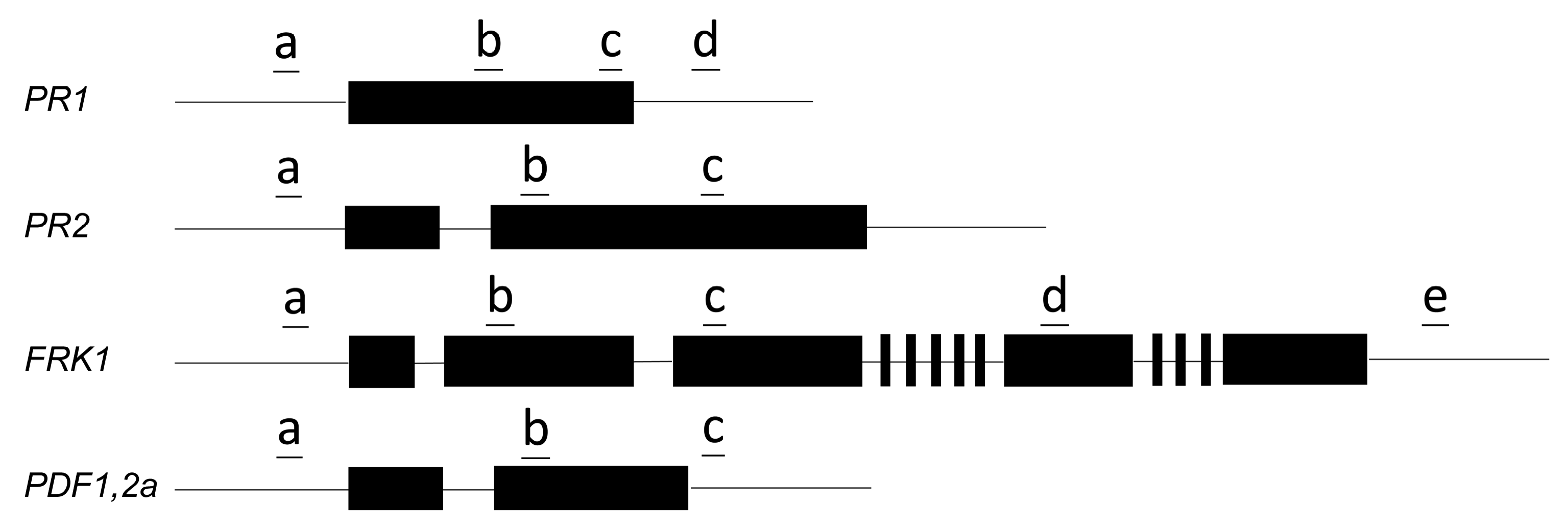
**

**Figure S5 |** Relative position of primers for ChIP-qPCR. Black boxes correspond to exons and “a” to “e” letters to amplified regions.

**
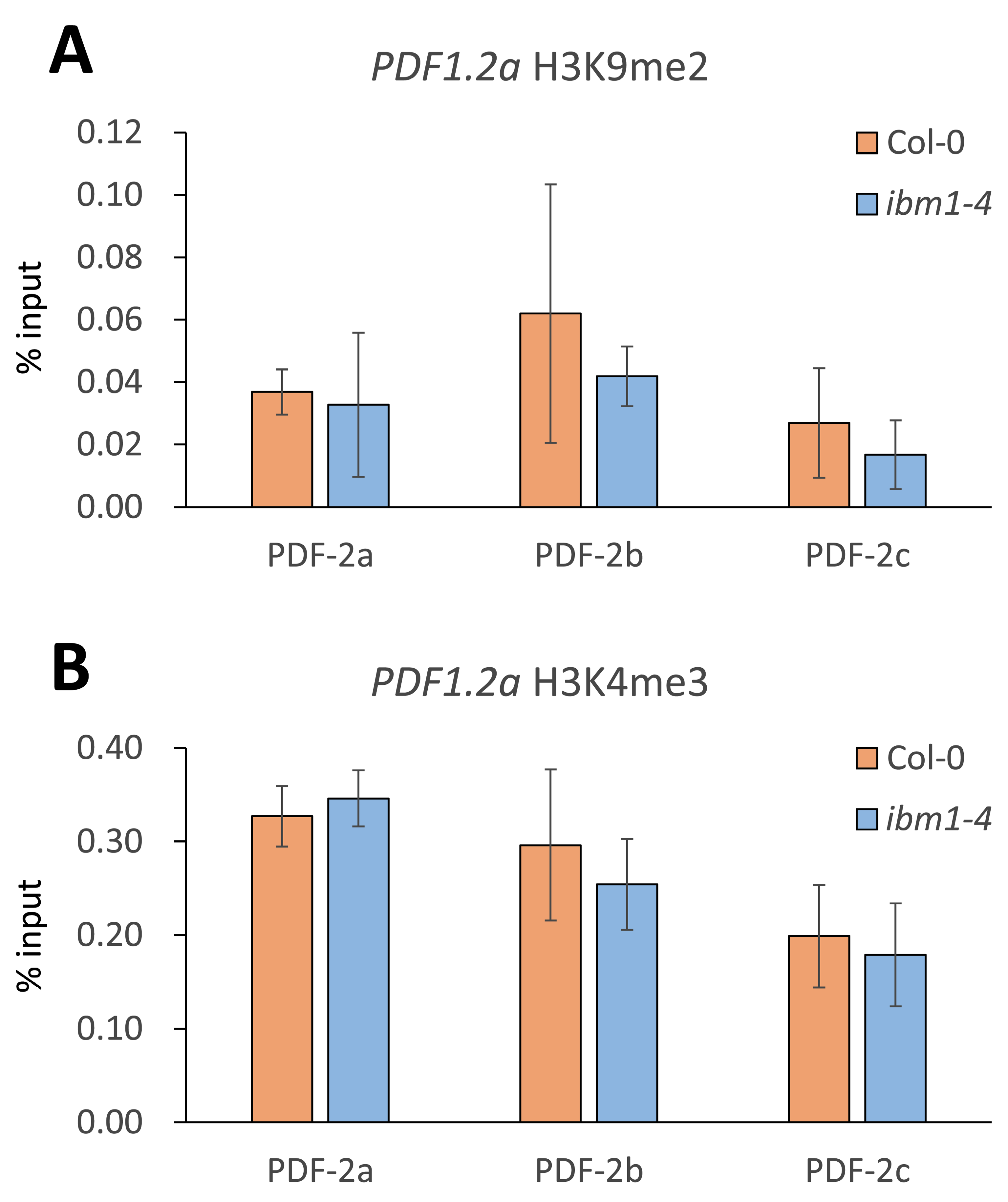
**

**Figure S6 |** The chromatin state of *PDF1.2a* is unaffected in the *ibm1-4* mutant. **(A)** Detection of H3K9me2 levels. Tissue of 14-day-old seedlings were pooled and ChIP was carried out using anti-H3K9me2 antibody for Col-0 and *ibm1-4*. The associated chromatin was quantified by qPCR with primers spanning across *PDF1.2a*. Primer sequences are listed in Table S3. Relative enrichment was calculated as percentage of input 2^-ΔCt^ (=2^-[Ct(ChIP)-Ct(Input)]^). Values represent average ± SD from 4 technical repeats (N = 4). The experiment was repeated twice with similar pattern and one representative repeat is shown. No significant differences were observed from Col-0 WT controls as determined by a paired two-tailed Student’s *t*-test (p < 0.05). **(B)** Detection of H3K4me3 level for *PDF1.2a* were evaluated and analyzed as in **(A)** with anti-H3K4me3 antibody.

**
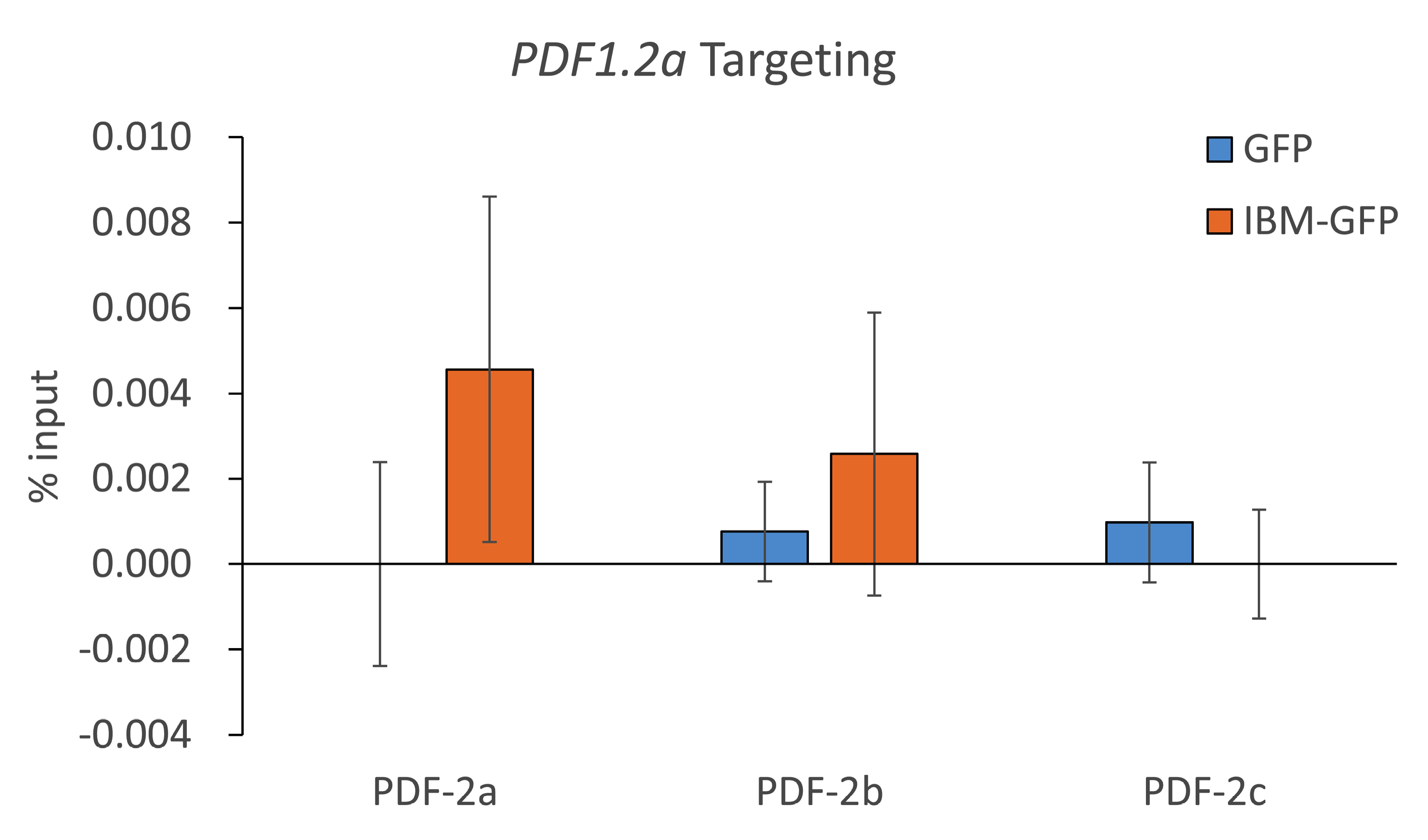
**

**Figure S7 |** IBM1 does not associate with the chromatin of *PDF1.2a*. Tissues of 14-day-old seedlings were pooled and ChIP was carried out using anti-GFP magnetic beads for *ProIBM1::IBM1-GFP* in the *ibm1-4* mutant background (IBM1-GFP) and GFP transgenic plants. The associated chromatin was quantified by qPCR with primers spanning across *PDF1.2a*. Relative enrichment was calculated as percentage of input 2^-ΔCt^ (=2^-[Ct(ChIP)-Ct(Input)]^). Values represent average ± SD from 4 technical repeats (N = 4). The experiment was repeated twice with similar patterns and one representative biological repeat is shown. No significant differences were observed from GFP controls as determined by a paired two-tailed Student’s *t*-test (p<0.05).


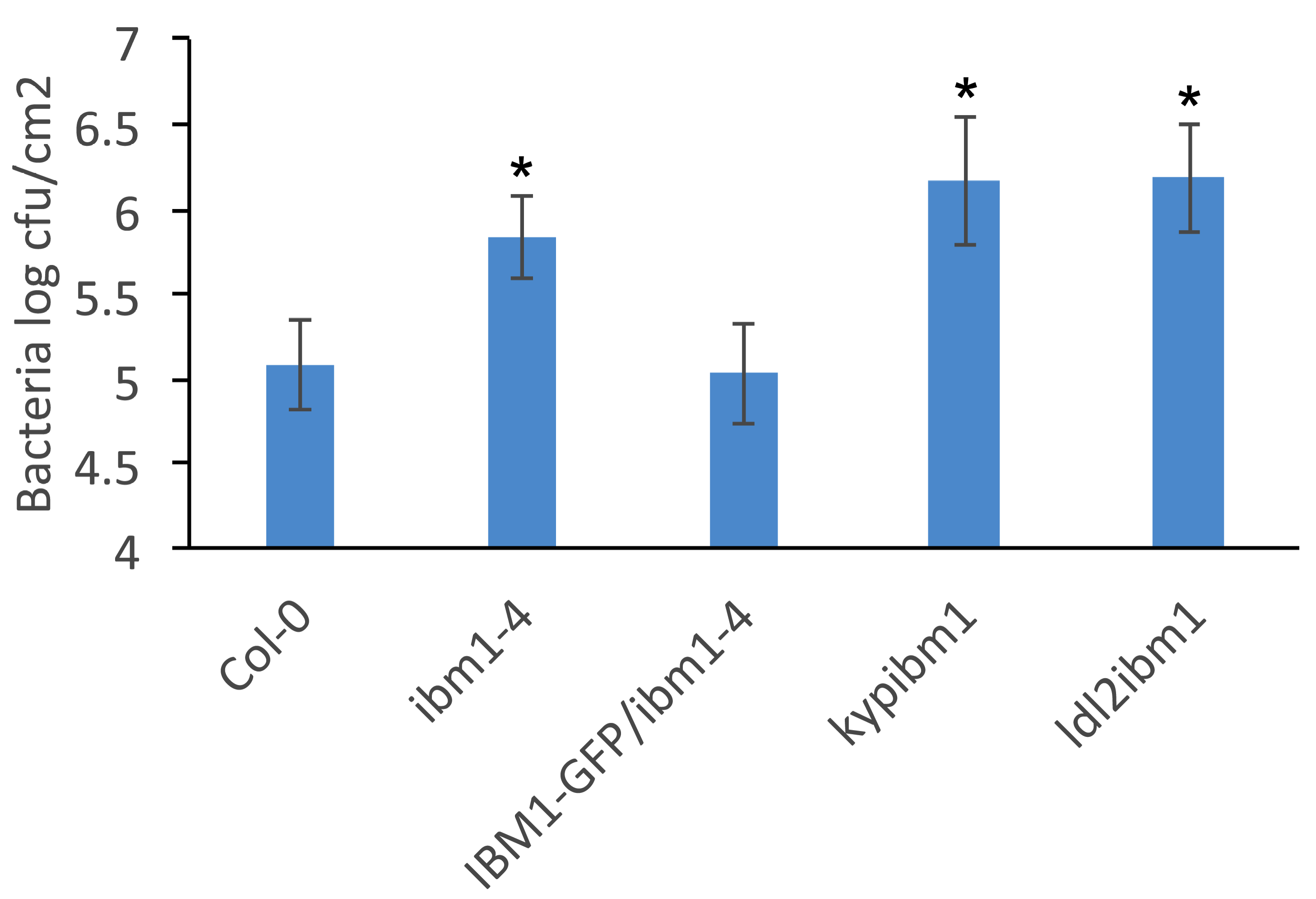


**Figure S8 |** Genetic repression of *ibm1*-induced immunity defects. Three leaves of 5-week-old plants were infiltrated-inoculated with 10^5^ cfu/mL *Pst* DC3000. Bacteria titers were evaluated at 3 dpi in Col-0, *ibm1-4*, IBM1-GFP/*ibm1-4*, *kypibm1* and *ldl2ibm1*. Values represent average ± SEM from 3 independent experiments each with at least 3 plants (N = 9). Asterisks indicate significant differences from the Col-0 WT as determined by a paired two-tailed Student’s t-test (*p < 0.05).

| **Table S1 \|** Primers for genotyping and *IBM1* gene expression. | |
| --- | --- |
| Primers | Sequences |
| LBb1.3 | ATTTTGCCGATTTCGGAAC |
| M1 | CGTTATTATTGGATATACCTGCATTAAT |
| M2 | CAAGCCATTAGATAAATACGAGTATAAG |
| M3 | CTTGTTGATTTCTACCTCAATGACTCT |
| M4 | ATCAAAACAGCATCACCAAGCTTCTGATT |
| F1 | GGGAGAAATCGATGGTGAGA |
| R1 | GGTCTGACACCGTTCAACAA |
| F2 | TGTTGAACGGTGTCAGACCT |
| R2 | CATGCATTTCTTGGCAACAT |
| AtACT2-F | GGTAACATTGTGCTCAGTGGTGG |
| AtACT2-R | AACGACCTTAATCTTCATGCTGC |
|  |  |
| **Table S2 \|** Primers for *PR1*, *PR2*, *FRK1* and *PDF1.2a* gene expression. | |
| Primers | Sequences |
| PR1-F | AAAACTTAGCCTGGGGTAGCGG |
| PR1-R | CCACCATTGTTACACCTCACTT |
| PR2-F | TGCAGAACATCGAGAACG |
| PR2-R | TACTCATCCCTGAACCTTCC |
| FRK1-F | GCCAACGGAGACATTAGAG |
| FRK1-R | CCATAACGACCTGACTCATC |
| PDF1.2a-F | AATCTTTGGTGCTAAATCGTGTG |
| PDF1.2a-R | CAACGGGAAAATAAACATTAAAA |
| UBQ10-F | GGCCTTGTATAATCCCTGATGAAT |
| UBQ10-R | AAAGAGATAACAGGAACGGAAACA |
|  |  |
| **Table S3 \|** Primers for ChIP assays. | |
| Primers | Sequences |
| PR1-aF | CCATGTATTTACAAAAACGTGAGA |
| PR1-aR | TTTGGGGTTCGTAAACATCG |
| PR1-bF | GGGTAGCGGTGACTTGTCTG |
| PR1-bR | CAAACTCCATTGCACGTGTT |
| PR1-cF | TCAGTGAGACTCGGATGTGC |
| PR1-cR | CGTTCACATAATTCCCACGA |
| PR1-dF | CATGCATACACACGTACATAAAGG |
| PR1-dR | TTCTCGTAATCTCAGCTCTTATTTG |
| PR2-aF | TGTGAATTATAATGGTGATTAAGGTGA |
| PR2-aR | GGTGCAACTGTGCAAGTCAG |
| PR2-bF | ATACCTTGCCAAGTCCATCG |
| PR2-bR | GATGTCAGAGCCACGGAGAG |
| PR2-cF | TCAAGGAAGGTTCAGGGATG |
| PR2-cR | AGATTCACGAGCAAGGGAGA |
| FRK1-aF | TCATTTGCCAAGGAAAAACC |
| FRK1-aR | TGTTCGGACCAGGACGTAAT |
| FRK1-bF | GATTCGGCGTTTGTTGATTC |
| FRK1-bR | TGCTTTGAGGGAAACTTCTCA |
| FRK1-cF | GCGAAACGAGAGCCTCTACC |
| FRK1-cR | TCTCGTCTGGTTGCTTTTCA |
| FRK1-dF | GAATGGGCCGTTGAAAACT |
| FRK1-dR | CCAAAACCTCCTTTGCCAAT |
| FRK1-eF | ACTTGCAAATGGTTGCTGAA |
| FRK1-eR | GGTGATGCTCGGGTAACTTG |
| PDF-aF | ATGTGTGGGGTTACCACGTT |
| PDF-aR | GAAATAAGCCAAAGATCAACGTCT |
| PDF-bF | AGTTGTGCGAGAAGCCAAGT |
| PDF-bR | GTTGCATGATCCATGTTTGG |
| PDF-cF | TCTTTGGTGCTAAATCGTGTG |
| PDF-cR | AACAACAACGGGAAAATAAACA |
